# Predictive Modeling of Healthcare Workers’ Priorities of the WHO 2030 Key Activities for Snakebite Prevention and Control in Ghana

**DOI:** 10.1155/jotm/7078381

**Published:** 2026-07-28

**Authors:** Eric Nyarko, Iddrisu Abugbil Atubiga, Fafa Shalom Tchorly, Nicholas Amani Hamman, Aashna Uppal, Nuhu Mohammed, Leonard Kyei, Eduardo Alberto Fernandez, Isaac Baidoo

**Affiliations:** ^1^ Department of Statistics and Actuarial Science, College of Basic and Applied Sciences, University of Ghana, Legon, Accra, Ghana, ug.edu.gh; ^2^ Department of Mathematics and Statistics, University of North Florida, 1 UNF Drive, Jacksonville 32224, Florida, USA, unf.edu; ^3^ Snakebite Treatment and Research Hospital, Kaltungo 770110, Gombe, Nigeria; ^4^ Nuffield Department of Medicine, The Global Health Network, Centre for Global Health and Tropical Medicine, University of Oxford, Oxford OX2 7SG, UK, ox.ac.uk; ^5^ Mathematics Applications Department, Wisconsin International University College, Legon, Accra, Ghana; ^6^ Department of Health Sciences, Brock University, St Catharines, Ontario, Canada, brocku.ca

**Keywords:** Ghana, machine learning model, maximum difference choice experiments, snakebites, WHO 2030 snakebite strategic key activities

## Abstract

Snakebite is a serious public health issue that causes a significant amount of morbidity and mortality. This has led the World Health Organization (WHO) to develop a roadmap to reduce snakebite envenoming (SBE) by half by 2030. This study predicted the activities healthcare workers consider most important among the 24 WHO SBE roadmap activities for preventing and controlling snakebites. The research involved healthcare workers and took place in two poor and mostly rural districts in the Eastern Region of Ghana. The study used traditional MaxDiff and five machine learning (ML) models to analyze the data. Healthcare workers prioritized “Making safe, effective antivenoms available, accessible, and affordable to all” as the most important activity, with a utility estimate (UE) of 0.8256 (95% confidence interval (CI): 0.7300 to 0.9213). This was followed by “Effective first aid care and ambulance transport” (UE = 0.4348, 95% CI: 0.3266 to 0.5430), “Coordinated data management and analysis” (UE = 0.3744, 95% CI: 0.2353 to 0.5134), and “Promoting advocacy, effective communication, and productive engagement” (UE = 0.3630, 95% CI: 0.2528 to 0.4732). Using choice experiments and ML models has helped reveal healthcare workers’ priorities regarding the WHO’s SBE roadmap activities. This approach gives a clear view of local opinions on key WHO activities, which is crucial for tackling the snakebite problem. By using this information in the broader health system, we can improve health outcomes and enhance the quality of life for snakebite patients in Ghana and other low‐ and middle‐income countries.

## 1. Introduction

About 5.8 billion people around the world are at risk of being bitten by venomous snakes. Each day, around 7400 people are bitten [1], leading to between 81,000 and 1,38,000 deaths each year. Nearly 4,00,000 survivors of snakebites face long‐term issues like blindness, tissue loss, amputations, and posttraumatic stress disorder [2]. In Northern Ghana, there are 56.4 snakebite cases for every 1,00,000 people, with a death rate of 1.35 per 1,00,000 [3]. In the Volta Region, local health data show 15.8 cases per 1,00,000 people and a death rate of 0.4% [4].

As efforts progress toward achieving the objectives of UHC2030, immediate action is essential to alleviate the suffering of the world′s most disadvantaged communities [5]. Recognizing the critical public health issue of snakebite envenoming (SBE), the World Health Organization (WHO) classified it as a Category A neglected tropical disease (NTD) in 2017 [6]. A resolution at the 2018 World Health Assembly [7] led to the development of a global roadmap with four strategic objectives, each with six key/operational activities to help reduce mortality and disability from SBE by half by 2030 [5]. Achieving this goal relies heavily on strong research evidence to help shape effective policies and programs [8, 9]. This need extends to creating and implementing plans relevant to our local communities to address SBE [1]. Therefore, rigorous and innovative research is necessary to inform the development of such localized plans and policies. These initiatives will help identify regional priorities that shape local snakebite research agendas and develop interventions that address community needs. The cutting‐edge technology of artificial intelligence (AI) holds significant potential in this area of tropical disease management [10], offering hope for the future of SBE management.

Many studies have used AI models to identify and classify snakes and to predict snakebites [11–15], but they do not incorporate statistical experimental designs. Combining AI or machine learning (ML) models with statistical experimental design can provide valuable insights to improve data‐driven decision‐making [16]. This study is innovative as it explores advanced maximum‐difference choice‐experiment techniques and integrates AI/ML applications into snakebite research.

Despite growing interest in rigorous and innovative research evidence to guide effective policies and programs for snakebite prevention and control, important data‐driven evidence gaps remain regarding stakeholder priorities for individual WHO roadmap activities, particularly in snakebite‐endemic countries where healthcare resources are constrained, and implementation decisions require careful prioritization. A previous study conducted in Ghana used a qualitative approach to rank the 24 WHO SBE operational activities for snakebite prevention and control [8]. This study, conducted in two poor and mostly rural districts [17, 18] in the Eastern Region of Ghana, was prompted by reported cases of snakebites and related fatalities [19, 20]. It contributes to the existing literature by offering quantitative, evidence‐based insights from AI/ML that align with the WHO’s operational activities. While our prior study quantified healthcare workers’ priorities regarding the WHO four strategic objectives for snakebite prevention and control in Ghana [21], it did not formally examine the 24 operational activities embedded within those objectives. Additionally, our earlier research focused on challenges related to antivenom availability, accessibility, utilization [22], and antivenom prioritization [23], but it did not address the broader implementation activities outlined in the WHO roadmap. By focusing on operational implementation priorities rather than broad strategic objectives, this study provides more granular and actionable evidence to guide policy formulation, implementation planning, and resource allocation for snakebite prevention and control. Therefore, the objective of this study was to predict healthcare workers’ priorities for the 24 SBE roadmap activities using a maximum‐difference choice experiment integrated with ML modeling approaches. By combining preference elicitation methods with ML algorithms, this study provides operational‐level evidence to support the implementation of the WHO SBE strategy in Ghana and other snakebite‐endemic settings.

## 2. Methods

### 2.1. Study Design

This cross‐sectional study focused on snakebites, a serious issue in the Kwahu Afram Plains North and South Districts of Ghana’s Eastern Region [19, 20]. It took place from August to December 2024, as reported in our previous study [21]. We used a multistage sampling method to select healthcare workers who treat snakebite patients in these areas. We also wanted to understand their priorities on the 24 activities outlined in the WHO snakebite strategy.

### 2.2. Designed Maximum Difference Statistical Choice Experiment

This study employed a maximum difference choice experiment approach, an advanced method for assessing preferences for various goods and services [24, 25]. A major benefit of this method is that it allows respondents to compare items directly [25, 26] rather than rank or rate them [8]. Experimental design is crucial for generating choice sets used in maximum difference studies. A well‐structured statistical experimental design helps identify the specific choices for the task, which are defined by a number of attributes and their corresponding levels. This method allows for effective manipulation of attributes and levels, enabling thorough testing of research objectives and instilling confidence in the study’s methodology. There were 1296 experimental conditions based on the WHO global roadmap with four strategic objectives, each with six operational activities. However, creating choice sets of size three from these experimental conditions would have resulted in an impractically large number of scenarios. To ensure the study’s efficiency and to prevent information overload for the respondents, we utilized combinatorial methods, including fractional factorial designs, incomplete block designs, and Hadamard matrices, to condense the scenarios into 12 choice sets of size three required for a best‐worst scaling task. We assume that these choice sets have an equal chance of being selected. In these effects‐type coded choice sets, the frequency of the first attribute’s six levels (L1 to L6) appeared as follows: L1 = 6, L2 = 6, L3 = 5, L4 = 8, L5 = 6, and L6 = 5. The frequency of the levels of the second attribute appeared as L1 = 5, L2 = 7, L3 = 7, L4 = 6, L5 = 7, and L6 = 4. The frequency of the levels of the third attribute appeared as L1 = 7, L2 = 8, L3 = 4, L4 = 6, L5 = 5, and L6 = 6, while the frequency of the levels of the fourth attribute appeared as L1 = 8, L2 = 9, L3 = 5, L4 = 6, L5 = 5, and L6 = 3. Table 1 shows a sample choice set.

**TABLE 1:** Example of one of the scenarios as it appeared in the survey.

WHO snakebite strategic objectives and key activities	Option A	Option B	Option C
Ensure safe and effective treatment	Investing in innovative research on new therapeutics	Integrated healthcare worker training and education	Better control and regulation of antivenoms

Empower and engage communities	Research on sociocultural and economic factors affecting outcomes	Accelerate development of prehospital treatments	Effective first aid and ambulance transport

Strengthen health systems	Enhanced disease burden monitoring and surveillance	Include snakebite in national and subnational health plans	Enhanced disease burden monitoring and surveillance

Partnership, coordination, and resources	Establish a strong and sustainable investment case	Establish a strong and sustainable investment case	Coordinated data management and analysis

I like this option MOST: *Tick one box only*	Option A [ ]	Option B [ ]	Option C [ ]

I like this option LEAST: *Tick one box only*	Option A [ ]	Option B [ ]	Option C [ ]

*Note:* Twelve scenarios of size three were generated for this study, and the sample scenario represents scenario number nine.

### 2.3. Sample Size and Data Collection

In this study, the sample size was calculated using the available scenarios and a proposed formula [27]. The computation indicated that a minimum of 128 respondents was necessary for our study. To enhance statistical power, we chose to gather data from 137 respondents, as similarly reported in our prior study [21]. We tested the survey tool in a pilot study with 20 healthcare professionals to ensure it was clear. During the survey, respondents were presented with 12 choice sets and tasked to select their most and least prioritized needs regarding the 24 WHO SBE operational activities. Participants documented their priorities by hand, using paper and a pencil. Two enumerators entered the data using Microsoft Excel before they were imported into JMP Pro (Version 17.0) for formal analysis.

### 2.4. Data Preparation for Model Building

There were no missing values, as data collection included real‐time validation checks by enumerators to ensure a complete dataset [21]. To improve our model’s performance and avoid overfitting, we split the data into two parts: 70% for training and 30% for validation. In this study, the predictor variables relate to the 24 operational activities from the WHO SBE roadmap and were treated as generic attributes. The response variable was treated as a continuous measure. We analyzed the data using traditional MaxDiff and 5 ML models. To compare these models, we examined several key metrics. We considered a predictor variable statistically significant if the *p* value was 0.05 or less or if the 95% confidence intervals (CIs) did not include zero. All statistical analyses were conducted using JMP Pro, Version 17.0.

## 3. Results

### 3.1. Participant Characteristics

Participating in this study were 137 individuals from a variety of healthcare positions, including community health nurses, pharmacists or dispensing technicians, physician assistants, clinical officers or medical doctors, and certified, enrolled, or general nurses, as reported in our prior study [21].

### 3.2. Model Fit and Overall Performance

Table 2 compares the traditional MaxDiff and ML models used in this study based on various evaluation metrics. The traditional model had the highest AICc and BIC values, which were 5240.0476 and 5347.6278, respectively, indicating a poorer model fit. In contrast, the LASSO model achieved the lowest AICc (3426.8732) and BIC (3537.5352). Although fitting the LASSO model took the longest time at 4945 ms, it demonstrated the best predictive performance, with an RASE of 0.7589095 and a negative log‐likelihood of 1692.1198. The elastic net regression model followed closely, with an AICc of 3426.8841, a BIC of 3537.5461, an RASE of 0.7589097, a negative log‐likelihood of 1692.1252, and a better fitting time of 4022 ms compared to the LASSO model. The generalized regression (forward selection) model was the most efficient, taking a fitting time of just 224 ms, while the pruned forward selection model took 601 milliseconds. Still, they had the highest AICc of 3431.9669 and an RASE of 0.7596380. In conclusion, while all the ML models are more efficient than the traditional model at capturing underlying data patterns and avoiding overfitting, the LASSO model is the best choice, achieving the lowest RASE, AICc, and BIC values, making it ideal for applications.

**TABLE 2:** Model performance evaluations.

Method	AICc	BIC	RASE	Elapsed time (ms)	Negative log‐likelihood
LASSO	3426.8732	3537.5352	0.7589095	4945.0000	1692.1198
Generalized regression model (forward selection)	3431.9669	3547.8678	0.7596380	224.0000	1693.6361
Generalized regression model (pruned forward selection)	3431.9669	3547.8678	0.7596380	601.0000	1693.6361
Elastic net	3426.8841	3537.5461	0.7589097	4022.0000	1692.1252
Ridge	3427.1392	3537.8012	0.7589715	3208.0000	1692.2527
MaxDiff model^∗^	5240.0476	5347.6278			

*Note:* AICc: corrected Akaike information criterion.

Abbreviations: BIC, Bayesian information criterion; LASSO, least absolute shrinkage and selection operator; RASE, root average squared error.

^∗^Traditional mode.

### 3.3. Prediction Profilers for Each Type of Model

**FIGURE 1:**
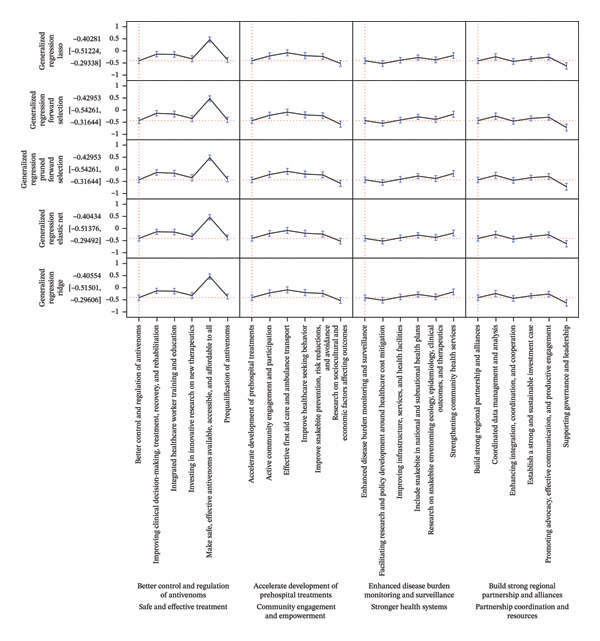
Prediction profiler for WHO SBE roadmap activities.

Figure 1 visually represents the utility profilers for each type of ML model. These profilers are essential in demonstrating the importance assigned to each of the 24 WHO SBE roadmap activities. The vertical red line in the utility profiler indicates the current value [21] of the operational activities, while the horizontal red lines show the current predicted values with their respective CIs of each response variable based on those operational activities. The ML models consistently identified the 24 roadmap activities as significant strategies for preventing and controlling SBE, with a few exceptions. Specifically, the key activities “Better control and regulation of antivenoms,” “Investing in innovative research on new therapeutics,” and “Include snakebite in national and sub‐national health plans” were not identified as significant strategies. However, it is noteworthy that “Include snakebite in national and subnational health plans” was recognized as a significant strategy by the generalized regression model (specifically using pruned forward selection and forward selection methods).

### 3.4. LASSO Regression Model

We presented the results from the LASSO regression model because it exhibited the best overall performance among all the candidate models. Utility estimates (UEs) based on 20 key activities alongside the WHO SBE four strategic objectives are presented in Table 3. It is worth noting that the key activities “Prequalification of antivenoms,” “Research on sociocultural and economic factors affecting outcomes,” “Strengthening community health services,” and “Supporting governance and leadership” were treated as reference levels. Among the 24 operational activities, the model identifies “Make safe, effective antivenoms available, accessible, and affordable to all” as the most important (UE = 0.8256, 95% CI: 0.7300, 0.9213) relative to the reference levels. Other WHO SBE roadmap activities of high importance include “Effective first aid care and ambulance transport” (UE = 0.4348, 95% CI: 0.3266, 0.5430), followed by “Coordinated data management and analysis” (UE = 0.3744, 95% CI: 0.2353, 0.5134), “Promoting advocacy, effective communication, and productive engagement” (UE = 0.3630, 95% CI: 0.2528, 0.4732), “Improve healthcare‐seeking behavior” (UE = 0.3182, 95% CI: 0.2123, 0.4242), “Active community engagement and participation” (UE = 0.3087, 95% CI: 0.2123, 0.4050), “Establish a strong and sustainable investment case” (UE = 0.2848, 95% CI: 0.1818, 0.3878), “Improve snakebite prevention, risk reduction, and avoidance” (UE = 0.2842, 95% CI: 0.1866, 0.3818), “Improving clinical decision‐making, treatment, recovery, and rehabilitation” (UE = 0.2302, 95% CI: 0.1354, 0.3250), “Integrated healthcare worker training and education” (UE = 0.2190, 95% CI: 0.1239, 0.3140), “Build strong regional partnerships and alliances” (UE = 0.2129, 95% CI: 0.1094, 0.3164), “Enhancing integration, coordination, and cooperation” (UE = 0.1856, 95% CI: 0.0811, 0.2902), and “Accelerate the development of prehospital treatments” (UE = 0.1095, 95% CI: 0.0304, 0.1886).

**TABLE 3:** LASSO regression model results for WHO SBE roadmap activities.

Attribute	Utility estimate	SE	Wald chi‐square	Prob > chi‐square	Lower 95%	Upper 95%
*Ensure safe and effective treatment:*						
Better control and regulation of antivenoms	−0.0388	0.0421	0.8493	0.3567	−0.1215	0.0438
Improving clinical decision‐making, treatment, recovery, and rehabilitation	0.2302	0.0483	22.6625	< 0.0001^∗^	0.1354	0.3250
Integrated healthcare worker training and education	0.2190	0.0485	20.3830	< 0.0001^∗^	0.1239	0.3140
Investing in innovative research on new therapeutics	0.0400	0.0568	0.4967	0.4809	−0.0713	0.1515
Make safe, effective antivenoms available, accessible, and affordable to all	0.8256	0.0487	286.3552	< 0.0001^∗^	0.7300	0.9213

*Empower and engage communities:*						
Accelerate development of prehospital treatments	0.1095	0.0403	7.3625	0.0067^∗^	0.0304	0.1886
Active community engagement and participation	0.3087	0.0491	39.4144	< 0.0001^∗^	0.2123	0.4050
Effective first aid care and ambulance transport	0.4348	0.0551	62.0828	< 0.0001^∗^	0.3266	0.5430
Improve healthcare‐seeking behavior	0.3182	0.0540	34.6708	< 0.0001^∗^	0.2123	0.4242
Improve snakebite prevention, risk reduction, and avoidance	0.2842	0.0498	32.5635	< 0.0001^∗^	0.1866	0.3818

*Strengthen health systems:*						
Enhanced disease burden monitoring and surveillance	−0.2150	0.0547	15.4199	< 0.0001^∗^	−0.3223	−0.1077
Facilitating research and policy development around healthcare cost mitigation	−0.3259	0.0559	33.9305	< 0.0001^∗^	−0.4355	−0.2162
Improving infrastructure, services, and health facilities	−0.1921	0.0491	15.2960	< 0.0001^∗^	−0.2884	−0.0958
Include snakebite in national and subnational health plans	−0.0835	0.0546	2.3407	0.1260	−0.1906	0.0234
Research on snakebite envenoming ecology, epidemiology, clinical outcomes, and therapeutics	−0.1766	0.0640	7.6010	0.0058^∗^	−0.3022	−0.0510

*Partnership, coordination, and resources:*						
Build strong regional partnerships and alliances	0.2129	0.0528	16.2518	< 0.0001^∗^	0.1094	0.3164
Coordinated data management and analysis	0.3744	0.0709	27.8461	< 0.0001^∗^	0.2353	0.5134
Enhancing integration, coordination, and cooperation	0.1856	0.0533	12.1224	0.0005^∗^	0.0811	0.2902
Establish a strong and sustainable investment case	0.2848	0.0525	29.3673	< 0.0001^∗^	0.1818	0.3878
Promoting advocacy, effective communication, and productive engagement	0.3630	0.0562	41.7115	< 0.0001^∗^	0.2528	0.4732

*Model fit statistics*						
AICc	3426.8732					
BIC	3537.5352					
RASE	0.7589095					
Negative log‐likelihood	1692.1198					

*Note:* AICc: corrected Akaike information criterion.

Abbreviations: BIC, Bayesian information criterion; RASE, root average squared error; SE, squared error.

^∗^Statistically significant attributes with *p* values ≤ 0.05 or exhibit 95% confidence intervals that do not include zero.

While statistically significant, participants tend to trade off certain key activities. These include “Facilitating research and policy development around healthcare cost mitigation” (UE = −0.3259, 95% CI: −0.4355, −0.2162), “Enhanced disease burden monitoring and surveillance” (UE = −0.2150, 95% CI: −0.3223, −0.1077), “Improving infrastructure, services, and health facilities” (UE = −0.1921, 95% CI: −0.2884, −0.0958), and “Research on snakebite envenoming ecology, epidemiology, clinical outcomes, and therapeutics” (UE = −0.1766, 95% CI: −0.3022, −0.0510).

## 4. Discussion

This study employed a novel combination of maximum‐difference choice experiments and ML modeling to quantify healthcare workers’ priorities regarding the key activities of the WHO 2030 SBE roadmap in Ghana. Unlike previous studies that examined broad strategic objectives [21], antivenom product prioritization [23], or barriers to antivenom availability, accessibility, and use [22], the present study focused on the 24 operational activities embedded within the WHO roadmap. Consequently, this study provides more granular evidence regarding which specific actions frontline healthcare workers believe should be prioritized to accelerate progress toward the WHO target of reducing snakebite‐related deaths and disabilities by 50% by 2030.

An important methodological finding was that all ML models outperformed the traditional MaxDiff approach, with the LASSO model demonstrating the best overall predictive performance. This finding suggests that regularized ML techniques may provide a useful framework for analyzing preference data generated through choice experiments, particularly when multiple correlated predictors are present. The integration of statistical experimental design with ML therefore offers an innovative analytical approach [16] for informing evidence‐based decision‐making in NTDs and other public health settings [10].

The most notable finding of this study was that healthcare workers overwhelmingly prioritized “Making safe, effective antivenoms available, accessible, and affordable to all.” This result is unsurprising given that antivenoms remain the only specific treatment for SBE and directly influence patient survival, recovery, and long‐term disability outcomes [1, 2, 28, 29]. In West Africa, snakebite continues to be substantially underrecognized despite causing considerable mortality, disability, and economic burden, particularly among rural populations with limited access to healthcare services [30]. Frontline healthcare workers routinely encounter the consequences of delayed treatment, inadequate antivenom supplies, financial barriers to access, and uncertainty regarding antivenom effectiveness. Their prioritization of antivenom access therefore reflects practical realities encountered in healthcare facilities rather than abstract policy preferences. A previous study conducted in Ghana has documented challenges related to the availability, accessibility, and utilization of antivenoms [22], highlighting persistent gaps in treatment delivery. Our findings suggest that healthcare workers view improving access to effective antivenoms as the single most important intervention for reducing the burden of SBE. This finding reinforces calls for strengthened antivenom procurement systems, sustainable financing mechanisms, improved distribution networks, and regional antivenom stockpiles to ensure uninterrupted access to treatment, particularly in rural and underserved areas [28, 29].

Another highly ranked priority was “Effective first aid care and ambulance transport.” This finding reflects the critical importance of the period immediately following a snakebite. In many rural communities, substantial delays occur between the time of envenoming and arrival at an appropriate healthcare facility. Delayed transportation often contributes to increased mortality, severe complications, prolonged hospitalization, and permanent disability related to SBE [31–33]. The prioritization of first aid and emergency transport indicates that healthcare workers recognize that improving survival requires interventions extending beyond hospital‐based treatment. Strengthening community awareness of appropriate first‐aid practices, expanding emergency referral systems, improving ambulance availability, and reducing geographical barriers to care may substantially improve clinical outcomes [33, 34]. This finding also highlights the importance of integrating community‐based interventions with facility‐based snakebite management strategies.

The emergence of “Coordinated data management and analysis” as one of the highest priorities provides important insights into existing health system challenges. Effective surveillance systems are essential for understanding disease burden, identifying high‐risk populations, monitoring intervention effectiveness, and informing resource allocation [35]. However, SBE remains substantially underreported in many low‐ and middle‐income countries [36]. Healthcare workers appear to recognize that weaknesses in surveillance and data systems limit researchers’ and policymakers’ ability to accurately estimate disease burden and effectively plan interventions. The prioritization of coordinated data management suggests that investments in surveillance infrastructure, standardized reporting systems, digital health technologies, and integration of snakebite indicators into national health information systems may be critical components of successful roadmap implementation. A great initiative is the addition of SBE data to the Global Health Observatory. This will help improve the quality and comparability of the data by creating standard clinical criteria that fit regional needs and a minimum set of data definitions for both community‐acquired and hospital‐acquired cases. These efforts will support better decision‐making [5, 29].

The prominence of activities related to advocacy, communication, healthcare‐seeking behavior, community engagement, and snakebite prevention highlights the continued importance of social and behavioral determinants of snakebite outcomes. Community misconceptions, reliance on traditional treatments, delays in seeking medical care, and poor awareness of appropriate first‐aid practices remain significant barriers to effective snakebite management in many endemic settings [3, 8, 34]. Healthcare workers appear to recognize that achieving meaningful reductions in snakebite morbidity and mortality requires active community participation alongside improvements in clinical care. These findings support WHO recommendations that emphasize community engagement, health promotion, risk‐reduction strategies, and culturally appropriate communication campaigns as essential components of comprehensive snakebite control programs [5, 29, 34].

A striking finding was that participants tended to trade off among several activities within the strategic objective of strengthening health systems, including disease surveillance, healthcare cost mitigation, infrastructure improvement, and research. Although these activities remain important, their lower UEs suggest that healthcare workers may perceive them as indirect or longer‐term investments compared with interventions that produce immediate clinical benefits. Frontline providers often operate in resource‐constrained environments where urgent patient needs dominate decision‐making. Consequently, interventions that directly improve treatment access, emergency response, and patient outcomes may be viewed as higher priorities than broader system‐level reforms. Nevertheless, it is important to emphasize that sustainable snakebite control ultimately depends upon strong health systems capable of supporting surveillance, research, financing, workforce development, and service delivery [1, 28].

The findings have several implications for policy and practice. First, national and district‐level health authorities should prioritize ensuring reliable access to effective and affordable antivenoms in snakebite‐endemic regions [1, 22, 29]. Second, investments in emergency transport systems, referral networks, and community first‐aid education should be strengthened to reduce treatment delays [31–34]. Third, improved surveillance systems and data management platforms are needed to generate reliable evidence for planning, monitoring, and evaluation [5, 29, 35]. Fourth, community engagement strategies should be expanded to improve healthcare‐seeking behavior, reduce harmful traditional practices, and increase awareness of prevention measures [3, 8, 34]. Finally, policymakers should consider incorporating healthcare workers’ perspectives into national snakebite strategies, as frontline providers possess valuable insights regarding implementation priorities and service delivery challenges.

This study contributes to the growing literature on SBE by extending previous research beyond broad strategic objectives and antivenom‐specific issues to examine the complete set of WHO roadmap implementation activities. While earlier studies identified which strategic objectives should receive greater attention [21], explored healthcare workers’ priorities using a qualitative approach to rank the operational activities [8], or examined challenges surrounding antivenom access and utilization [22], the present study rigorously provides operational‐level evidence on the specific activities that healthcare workers believe should be prioritized. This level of detail is particularly valuable for policymakers and program implementers seeking to translate the WHO roadmap into practical action at national and subnational levels.

It is important to acknowledge the study’s limitations. The study was conducted in two districts within Ghana and therefore may not fully capture the perspectives of healthcare workers in other geographical settings. The study also focused exclusively on healthcare workers and did not include community members, policymakers, traditional healers, researchers, and other stakeholders involved in snakebite prevention and control. Furthermore, the study elicited stated preferences rather than directly observing clinical practices or implementation experiences. Future research should use a mix of methods, combining quantitative exercises to set priorities with in‐depth investigations and field assessments of actual snakebite management practices. Such studies would enable comparisons between what healthcare workers identify as priorities and what occurs in routine practice.

In conclusion, this study provides quantitative evidence regarding healthcare workers’ priorities for implementing the WHO 2030 SBE operational activities in Ghana. The findings demonstrate that improving antivenom access, strengthening emergency response systems, enhancing data management, and promoting community engagement are considered the most critical actions to reduce the burden of SBE. By providing operational‐level priorities rather than broad strategic preferences, this study offers actionable evidence that can support policy development, resource allocation, implementation planning, and future research aimed at achieving the WHO target of halving snakebite‐related mortality and disability by 2030.

## Author Contributions

All authors have a priceless contribution to the success of this study. Eric Nyarko: conceptualization, methodology, software, validation, formal analysis, resources, investigation, data curation, writing–original draft, writing–review and editing, and visualization. Iddrisu Abugbil Atubiga: formal analysis, investigation, data curation, project administration, and writing–review and editing. Fafa Shalom Tchorly: writing–review and editing and visualization. Nicholas Amani Hamman and Aashna Uppal: writing–review and editing, visualization, and validation. Nuhu Mohammed and Eduardo Alberto Fernandez: writing–review and editing, and visualization. Leonard Kyei: writing–review and editing, and validation. Isaac Baidoo: conceptualization, writing–review and editing, and validation.

## Funding

The authors received no specific funding for this work.

## Disclosure

A preprint version of this manuscript was previously made available on medRxiv [37].

## Ethics Statement

The study received ethical approval from the Ghana Health Service Ethics Review Committee (GHS‐ERC073/04/24). The study protocol was conducted according to the Declaration of Helsinki and adhered to all ethical guidelines and regulations. After explaining the purpose of the study to all participants, written informed consent was obtained. Participants were informed that their participation was voluntary and that they had the option to choose whether to participate in the study or not.

## Consent

Please see the Ethics Statement.

## Conflicts of Interest

The authors declare no conflicts of interest.

## Data Availability

Deidentified individual data supporting the results can be shared only if the investigator has approval from an Institutional Review Board, Independent Ethics Committee, or Research Ethics Board, as applicable. Additionally, they must execute a data use and sharing agreement with the Ghana Health Service (GHS). To initiate this process, the Principal Investigator should submit a request to the Ethical and Protocol Review Committee of GHS at ethics.research@ghs.gov.gh to begin the data use/sharing agreement.
